# Common Errors in Diagnosis: Errors in Clinical Pathology
*A Lecture delivered at the Polyclinic.


**Published:** 1913-11-22

**Authors:** V. H. Wyatt Wingrave


### COMMON ERRORS IN DIAGNOSIS.

### Errors in Clinical Pathology.*

By V. H. WYATT WINGRAVE, M.D.

The errors made with the microscope and the test-tube are, in their way, as numerous as those made with stethoscope, laryngoscope, and other instruments of precision. But he who practises pathology has the great advantage that he can confirm or check his results as he proceeds; this he should always do before arriving at and stating a dogmatic decision. The work must be practical; it is most misleading to be guided merely by statements made in books, particularly translated epitomes from foreign literature, which are very treacherous. The mere misplacing of a decimal point may cause endless trouble, and the translator does not always hit upon the equivalent English expression or term.

With regard, first, to urine testing. When you have a turbid specimen of urine, it is not easy to say at first what the turbidity is due to. Is the turbidity permanent or only temporary? It may become clear on cooling after being passed; and, if so, the turbidity has usually been due to the presence of phosphates. But it may be clear when first passed, and become opaque on standing. That is likely to be due to urates. You can thus fairly exclude organic change. If the turbidity does not disappear on warming, we go a little farther, and add alkali to it. If it does not disappear it is not a case of urates. If you add acid and it does not disappear, the turbidity is not due to earthy phosphates. If you add a drop of liquor potassæ to it it becomes fairly clear and transparent; but as you pour it from one vessel to another you notice it has become gelatinous—what some call "ropy." That is said to be diagnostic of the presence of pus. But that is only partially true; you must not diagnose it from that reaction alone. But it may remain watery and transparent, and may mean there is mucine. If, on adding acid, there is marked flocculence, that is somewhat confirmatory; at all events, it excludes a good deal. Take a film or sediment, and if there is not much sediment, centrifuge; after letting it stand examine for cells and for bacteria. Sometimes the turbidity is not evenly distributed through the whole specimen; then, if it does not disappear on heating and is not altered by the addition of acid or alkalies, suspect bacteria.

With regard to testing urine for albumen, nothing can be more simple than this reaction; but perhaps none is more productive of misunderstanding. We habitually use heat or nitric acid—cold or hot—but neither is reliable. People will say, "You need not trouble to examine for albumen, there is plenty." But proper examination does not reveal a trace of it. Nitric acid causes a precipitate, but it soon disappears. The first evidence of albumen must be accepted with doubt, particularly if the response is a striking one. The reason is that albumoses give the reaction, and these may be present in the urine of any healthy person, particularly if he has been exercising. It is also found in the urine of pregnant women, and sometimes has led to a grave view being taken of the case, visions of eclampsia rising in the mind of the practitioner, whereas it is a simple peptone, and has no serious import whatever. You must confirm your nitric-acid test by reagents which never lead you astray. I depend most upon sulphanilic acid and tartaric acid. When the first of these is added, and the mixture heated, the albumen is intensified, but an albumose is redissolved. Albumen is sometimes found in the urine in cases of fever. If a case is suspected to be diphtheria, test the urine, for albumen may appear very early indeed. But remember that albumoses may occur in any condition where there is a temperature disturbance. Sub-febrile conditions in children cause a great deal of anxiety.

You may have a specimen of urine containing sugar. You ask if it is glycosuria, or is it simply functional disturbance? These latter troubles seem to occur mostly about July and August—namely, when stone-fruit is most abundant. The sugar found is generally pentose, and this and sugars of this class give a reaction much like that of glucose. There is difficulty in distinguishing one from the other. Pentose is generally found in quite small quantities, whereas glucose is often present in large amount; and if it is pentose, the next specimen you examine from the patient may not show any. So do not be satisfied with examining only one sample. If the sugar occurs in all, you have very likely to deal with something more important than diet disturbance. In true glycosuria there is generally some profound change in the blood. In well-established diabetes you will generally find small traces of sugar in the blood. Nothing is more difficult in chemistry than to examine for sugar in a specimen in which there is hæmoglobin and protein, the usual constituents of the blood. So we must try to get a simple test as to whether a patient is a true diabetic or not. Take a couple of test-tubes. Prick the patient's thumb and allow a small amount of blood from it to trickle down the test-tube. Mix with it a very weak solution of methylene blue. (You can use the test for cerebro-spinal fluid as well as blood.) Add an equal part of liquor potassæ. This gives a solution you can just see through. Divide it into two parts. Into one put a drop of your own blood, and into the other some of the patient's blood. I have here a specimen of blood which contains sugar and a specimen which does not. On heating that containing sugar the colour entirely disappears, whereas in the other the colour is not affected, though there may be a little alteration in density, owing to the hæmoglobin. This

*A Lecture delivered at the Polyclinic.

enables you to detect the smallest quantity of sugar in blood, cerebro-spinal fluid, or nasal flow; and you can thus say whether a patient is the subject of diabetes.

Then there is the question of acetone. Acetonuria used to be regarded as a very serious affair; indeed it was said to herald the post-mortem room. But it is only of importance when it is present in combination with sugar. Acetone in the urine may be due to errors of diet; it may be present in tuberculosis and a number of other conditions. There are some difficulties in connection with detecting acetone in combination with sugar. In the urine creatinin is constantly present, but in varying amount. It is doubtless derived from creatin in the food, because after a heavy meat meal creatinin will appear in the urine. Creatinin gives the same reaction as does acetone. Add to the urine a few drops of nitro-prussi, and to that add liquor potassæ, and you get a striking red colour. Sometimes this colour will come out vigorously, sometimes scarcely at all. When acetone gives that test some other means must be adopted, and I think the best is the iodoform test.

It is rare to find urine alkaline unless that alkalinity is due to ammonia, and that you can smell. The acidity may be so low as scarcely to give the ordinary reactions; you get a blue reaction on red litmus, and a red reaction with blue litmus. Is it acid or is it alkaline? And if acid, how much acidity is there? That can only be done by titration; you know how much acidity is present by the amount of titration solution you have used. Employing urotropine in a case of suspected bacteriuria is useless unless there is some acid present. To elaborate the formalin there must be an acidity over 5.

With regard to blood, perhaps the most serious mistakes are those made in the search for occult blood—from the bowel, for instance. I prefer the benzidine test. Grind the fæces up and filter, then examine the filtrate for blood. On applying a solution of benzidine and ozonic ether there is a precipitate. If there is blood you will see in a few moments, first, a greenish tinge, which afterwards deepens into azure blue. You may mistake that for iron, but if there is any, even a trace of iron in the fæces, you will get a most striking blue reaction at once on adding the benzidine.

Unsound results can easily be obtained if the blood for examination is not collected in the right way. Many text-books say the blood should be collected from the lobule of the ear, but that is a mistake. The lobule contains much elastic tissue, which quickly closes up the puncture you have made, and hence you need to milk the ear, which does not give blood with its constituents in proper proportions. Collect the blood from the back of the thumb, between the nail and the first knuckle, after putting on a bandage above the knuckle. By reapplying the bandage and well swinging the arm between each extraction, as much as 5 c.c. of blood can be obtained from one thumb. And do not merely puncture with a needle, but with a lancet which has a cutting edge, and insert it obliquely. You will not then get serum instead of corpuscles. In making your film remember the corpuscles are of differing size, the leucocytes and myelocytes being biggest, and the smallest the lymphocytes, so that in making a spread you will move your leucocytes to the edge and the end of the film, while the lymphocytes, being the smallest, will remain in the middle. If you have not this in mind you are liable to diagnose lymphocytosis from a quite normal film. You will generally find leucopænia, or a deficiency of leucocytes, in typhoid, influenza, and measles. The reverse of that, or leucocytosis, is found in pneumonia, diphtheria, and scarlet fever. There may be great difficulty in the early stages in distinguishing between typhoid fever and pneumonia, but the blood examination will put you right. Another important point is the presence of abnormal forms; one may easily mistake a lymphocyte for a normoblast or an erythroblast. You do not find a blast except in serious blood states, such as pernicious anæmia and polycythæmia. As a rule, in the erythroblast there is a distinct cytoplasm around the nucleus, and it will be the same colour as all the other red corpuscles.

If you have a bad case of plethora in a patient of about fifty years of age your diagnostic powers will be seriously tested. He gradually gets worse and lethargic, yet his pulse is very full. He has considerable dyspnœa and palpitation, and on examination you hear extraordinary musical murmurs. A blood film will help you a good deal. You want to know particularly whether there is an excess or diminution of hæmoglobin and red corpuscles. A piece of white filter-paper is practically all that you want. Take some blood from the patient's thumb and some from your own, and compare the two in a strong light, and if you find the patient's blood is richer in colour than yours you may be sure it is polycythæmia or erythrocythæmia. It seems as if the pulmonary circulation is interfered with, that the oxygen carrying is insufficient, and large numbers of red corpuscles are produced for which there is not even standing room in the blood, and often it is richly charged with hæmoglobin.

Considerable attention has been drawn to the diagnosis of malignant disease by estimating the reaction of the blood. Its alkalinity is high in malignant disease. Examination in these cases shows complete absence of hydrochloric acid in the gastric juice. The proper time to test for this is two or three hours after a meal.

Two of the most important diseases to test for are tubercle and diphtheria.

In tubercle the error is often great. You examine the sputum and find it swarming with bacilli. These are acid-fast bacilli, but if you give them a bath of alcohol you will find the fuchsin stain is washed away: they are not alcohol-fast bacilli. Acid-fast bacilli are found in butter, cheese, atrophic rhinitis, the different varieties of ozæna. The bacilli are not tubercle unless they are alcohol-fast as well as acid-fast. On examination of pus which is escaping from a fistula you may find it is actinomycosis and not tubercle. Prominence has also been given lately to the presence of albumen in the sputum, but the statements being made are dangerous.

